# Using Implementation Science to Inform and Evaluate Health Policy

**DOI:** 10.1093/geroni/igab046.191

**Published:** 2021-12-17

**Authors:** Julie Bobitt, Beth Prusaczyk

**Affiliations:** 1 Center for Dissemination and Implementation Science, University of Illinois at Chicago, Illinois, United States; 2 Washington University School of Medicine in St. Louis, Saint Louis, Missouri, United States

## Abstract

Public health policies can be a tool for the promotion and protection of older adult’s well-being but how can we ensure that policies will be effective and applied as intended? This presentation will discuss how implementation science can be used to both inform and evaluate health policies. Scientific evidence developed by applying dissemination and implementation frameworks can be used to inform policy makers as they develop legislation. When used to evaluate policy, D&I frameworks can be applied to examine policy diffusion, how a state, community, or individual organization chooses to carry out the policy, and the impact that policy has on the intended population. D&I frameworks are an effective way to measure the difference between policy intent and what actually happens when a policy is implemented. Examples of how D&I frameworks have been used to inform and evaluate policy will be shared.

